# Characterization of Photo-Crosslinked Methacrylated Type I Collagen as a Platform to Investigate the Lymphatic Endothelial Cell Response

**DOI:** 10.3390/lymphatics2030015

**Published:** 2024-09-19

**Authors:** Brian N. K. Ruliffson, Stephen M. Larson, Eleni K. Xhupi, Diana L. Herrera-Diaz, Catherine F. Whittington

**Affiliations:** 1 Department of Biomedical Engineering, Worcester Polytechnic Institute, Worcester, MA 01609, USA; 2 Department of Biomedical Engineering and Chemical Engineering, University of Texas at San Antonio, San Antonio, TX 78249, USA

**Keywords:** methacrylated collagen, mechanical assessment, photoinitiator, human dermal lymphatic endothelial cells, disease modeling

## Abstract

Despite chronic fibrosis occurring in many pathological conditions, few in vitro studies examine how fibrosis impacts lymphatic endothelial cell (LEC) behavior. This study examined stiffening profiles of PhotoCol^®^—commercially available methacrylated type I collagen—photo-crosslinked with the photoinitiators: Lithium phenyl-2,4,6-trimethylbenzoylphosphinate (LAP), Irgacure 2959 (IRG), and Ruthenium/Sodium Persulfate (Ru/SPS) prior to evaluating PhotoCol^®^ permeability and LEC response to PhotoCol^®^ at stiffnesses representing normal and fibrotic tissues. Ru/SPS produced the highest stiffness (~6 kilopascal (kPa)) for photo-crosslinked PhotoCol^®^, but stiffness did not change with burst light exposures (30 and 90 s). The collagen fibril area fraction increased, and dextran permeability (40 kilodalton (kDa)) decreased with photo-crosslinking, showing the impact of photo-crosslinking on microstructure and molecular transport. Human dermal LECs on softer, uncrosslinked PhotoCol^®^ (~0.5 kPa) appeared smaller with less prominent vascular endothelial (VE)-cadherin (cell–cell junction) expression compared to LECs on stiffer PhotoCol^®^ (~6 kPa), which had increased cell size, border irregularity, and VE-cadherin thickness (junction zippering) that is consistent with LEC morphology in fibrotic tissues. Our quantitative morphological analysis demonstrates our ability to produce LECs with a fibrotic phenotype, and the overall study shows that PhotoCol^®^ with Ru/SPS provides the necessary physical properties to systematically study LEC responses related to capillary growth and function under fibrotic conditions.

## 1. Introduction

The lymphatic system has long been viewed as a passive component of the immune and circulatory systems. However, in the last 15 years more evidence has emerged to show that the lymphatic system plays an active role in wound healing, cancer, and fibrosis [1] through regulation and dysregulation of the vasculature. In many of these healing and disease states, the tissue surrounding lymphatic vasculature undergoes dynamic biochemical (e.g., soluble factors) and biophysical (e.g., stiffness) changes that influence cell behavior [2–5]. One important biophysical change is the increased tissue stiffening that is often associated with chronic fibrosis—a pathological wound healing condition marked by excess tissue deposition and scarring [6]. Increased tissue deposition and the subsequent tissue densification also alter tissue transport properties, which change the biochemical environment by altering how signaling molecules are produced and move through the tissue to reach cells. However, little is known about how tissue stiffening affects lymphatic vessel growth and function, particularly under disease conditions where tissue stiffness is much higher than in healthy tissue or during lymphatic development. Thus, it is important to leverage in vitro modeling approaches that use materials capable of exhibiting mechanical properties of healthy and fibrotic tissue environments to understand the impact of stiffness on lymphatic vasculature in fibrosis and wound healing.

Over the last several years, there has been more interest in investigating the relationship between tissue stiffness and lymphatic vasculature. To date, these studies primarily focus on lymphatic development and/or employ modeling strategies that approximate tissue stiffness levels observed across developmental and growth stages. Both Frye et al. [7] and Alderfer et al. [8] have demonstrated that Human Dermal Lymphatic Endothelial Cells (HDLECs) appear to be primed for lymphatic capillary formation on softer extracellular matrix (ECM) substrates of approximately 0.03–0.2 kilopascal (kPa). In these studies, HDLECs show increased expression for genes related to cell–matrix adhesion, cell migration (e.g., proteolytic enzymes), and new lymphatic vessel growth, while genes for cell proliferation were more likely to be downregulated. Collectively, they demonstrate the mechanosensing capabilities of HDLECs and highlight the importance of ECM stiffness in directing HDLEC behavior with stiffer substrates appearing to be more inhibitory to behaviors related to lymphatic capillary growth [7,8]. However, stiff matrices produced in vitro have the potential to represent stages of in vivo fibrosis where increased ECM stiffness plays a role in hindering lymphatic capillary growth (e.g., myocardial edema, secondary lymphedema) or supporting it (e.g., cancer, renal fibrosis).

As we consider materials for in vitro lymphatic studies, they should also reflect the ECM composition of tissues surrounding lymphatic vessels [4,7–9]. Substrate stiffness differences have been achieved using materials that support a range of stiffness levels but only for 2D monolayer cultures (i.e., Softwell^™^ and Softslip^™^ dishes [7]). Tunable hydrogels that support 2D or 3D cultures and have ECM composition relevant to lymphatic tissue interactions [10,11] (e.g., hyaluronic acid (HA) composites [8]) have also been used. Type I collagen is a viable ECM candidate for lymphatic studies due to its prominence in connective tissues and increased deposition and crosslinking in fibrotic tissues [12]. Lymphatic capillaries are also directly attached to collagen-rich ECM through adhesion molecules like integrins that bind collagens and fibronectin, as well as elastin microfibril interfacer 1 [13–15]. Therefore, fibrosis-mediated changes in type I collagen structure (e.g., crosslinking) and stiffness can be sensed by HDLECs. Methacrylated type I collagen can be used to generate matrices across a wide stiffness range with “on demand” photo-crosslinking (up to 8 kPa) [16,17], while maintaining the higher-order fibrillar microstructure found in in vivo tissue. However, despite methacrylated collagens being shown to have a robust mechanical response and support cell viability of multiple cell populations [16–19], they have not been leveraged for lymphatic endothelial cell (LEC) cultures.

In the current study, PhotoCol^®^, a methacrylated type I collagen formulation (Advanced BioMatrix, Inc., Carlsbad, CA, USA) was characterized on its “on demand” stiffening properties and permeability prior to serving as a substrate to assess the effects of ECM stiffness on HDLEC morphology. We hypothesized that PhotoCol^®^, crosslinked with one of its three commercially available photoinitiators, would increase stiffness to pathological levels while simultaneously decreasing its permeability. We also hypothesized that stiffer hydrogels would induce an LEC phenotype that was indicative of the fibrosis response (i.e., thicker, zipper-like cellular junctions). Of the three photoinitiators tested (Lithium phenyl-2,4,6-trimethylbenzoylphosphinate (LAP), Irgacure 2959 (IRG), and Ruthenium/Sodium Persulfate (Ru/SPS)), Ru/SPS yielded photo-crosslinked PhotoCol^®^ samples with the greatest dynamic stiffness range (0.5–6 kPa) with no stiffness difference upon burst light exposures. We then showed that photo-crosslinking also decreased permeability of 40 kilodalton (kDa) dextran but not 60–76 kDa dextran. Morphological assessment of HDLECs cultured on PhotoCol^®^ (with Ru/SPS) at high stiffness (6 kPa) showed thicker cell–cell junctions of vascular endothelial (VE)-cadherin) and increased average cell area and shape irregularity compared to soft hydrogels, which is consistent with morphological changes in lymphatic capillaries within fibrotic tissues. Overall, these results demonstrate that we can leverage on-demand stiffening of PhotoCol^®^, along with its fibrillar microstructure, as a substrate for in vitro models of lymphatic capillaries focused on modeling fibrotic disease states.

## 2. Results

### 2.1. PhotoCol^®^ Hydrogels with Ruthenium/Sodium Persulfate Achieve a Broad Dynamic Stiffness Range

There are multiple options for photoinitiators for methacrylated materials based on their irradiation wavelength, polymerization mechanisms, and stiffening capacities. We chose photoinitiators that use visible light to facilitate photo-crosslinking—Lithium phenyl-2,4,6-trimethylbenzoylphosphinate (LAP; 405 nm) and Ruthenium/Sodium Persulfate (Ru/SPS; 400–450 nm)—to avoid using ultraviolet (UV) light. These two photoinitiators also have better cytocompatibility than Irgacure 2959 (IRG; 365 nm), which uses UV light for photo-crosslinking. Irgacure was included because it is a common photoinitiator that has been previously characterized on its stiffness, degradation, and cytocompatibility [16].

**Figure 1.**
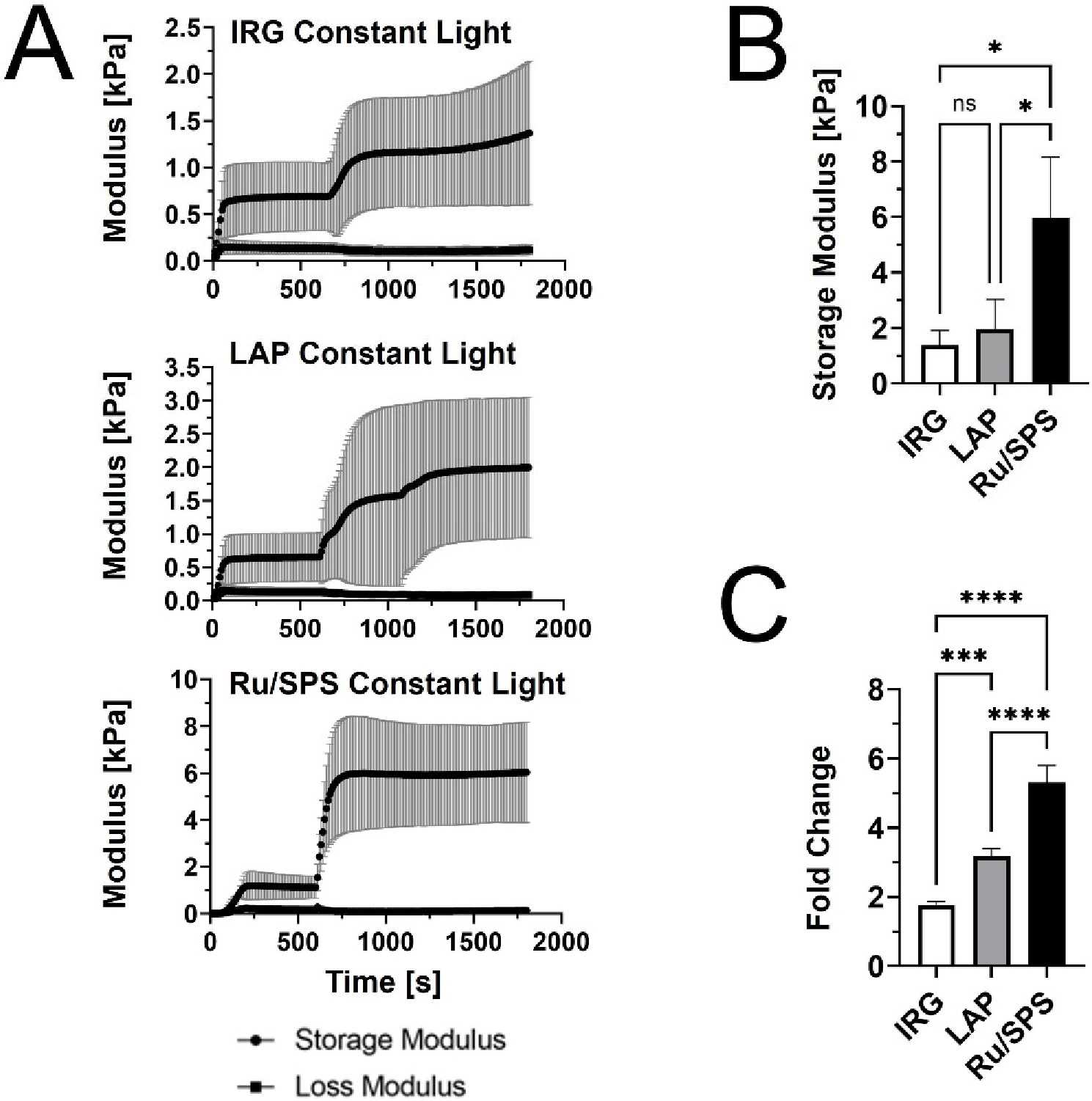
The viscoelastic properties of PhotoCol^®^ with photoinitiators and constant light exposure. (**A**) Shear storage modulus (G’) and shear loss modulus (G”) vs. time for PhotoCol^®^ combined with one of three photoinitiators: Irgacure (IRG), Lithium phenyl-2,4,6-trimethylbenzoylphosphinate (LAP), and Ruthenium/Sodium Persulfate (Ru/SPS). Samples self-assembled for 600 s (10 min) prior to photo-crosslinking with 365–405 nm light for 20 minutes (constant light exposure). Curves represent G’ and G” (mean ± SD; n = 3–4). (**B**) The maximum G’ calculated from G’ vs. time curves for PhotoCol^®^ samples with photoinitiators. Bars represent mean + standard deviation (SD) (n = 3–4). (**C**) Calculated fold change (mean + SD) before and after photo-crosslinking for PhotoCol^®^ samples with photoinitiators. Significance for (**B,C**) were determined with a one-way analysis of variance (ANOVA) and a Tukey’s post-hoc test (ns = no significance, * *p* < 0.05, *** *p* < 0.0001, and **** *p* < 0.00001).

We first performed rheological testing in oscillatory shear to compare the stiffening profiles of PhotoCol^®^ with IRG, LAP, and Ru/SPS under constant light (20 min total light exposure after self-assembly) (Figure 1A). Results showed that PhotoCol^®^ with Ru/SPS had a more predictable stiffening profile with a rapid increase in the shear storage modulus (G’) after photoinitiation (light exposure at 10 min after self-assembly) and flat, steady stiffness plateau before and after light exposure. The tests also showed that PhotoCol^®^ reaches the plateau of maximum stiffness with photo-crosslinking in approximately 3 min for samples containing IRG and Ru/SPS and approximately 6 min for samples containing LAP, and as expected, the shear loss modulus (G”) was much lower than G’ over the entire duration of the test. The Ru/SPS samples had significantly higher stiffness values than LAP and IRG (Figure 1B) at 5.93 ± 2.2 kPa for Ru/SPS compared to 1.96 ± 1.06 kPa and 1.38 ± 0.53 kPa for LAP and IRG, respectively. The higher stiffness values indicated that PhotoCol^®^ samples with Ru/SPS were more elastic and mechanically sound than samples with LAP or IRG. We also calculated the fold change increase in stiffness before and after photo-crosslinking to determine the dynamic range for each photoinitiator. All photoinitiators yielded significantly different dynamic ranges with the lowest being PhotoCol^®^ with IRG and the highest being PhotoCol^®^ with Ru/SPS (Figure 1C). Overall, the stiffness response for Ru/SPS, which had the highest maximum stiffness and greatest dynamic stiffness range, combined with the use of non-UV light (405–450 nm), informed our choice of using Ru/SPS as the photoinitiator for remaining studies.

### 2.2. Stiffness of PhotoCol^®^ with Ruthenium/Sodium Persulfate Is Unchanged with Burst Light Exposure

**Figure 2.**
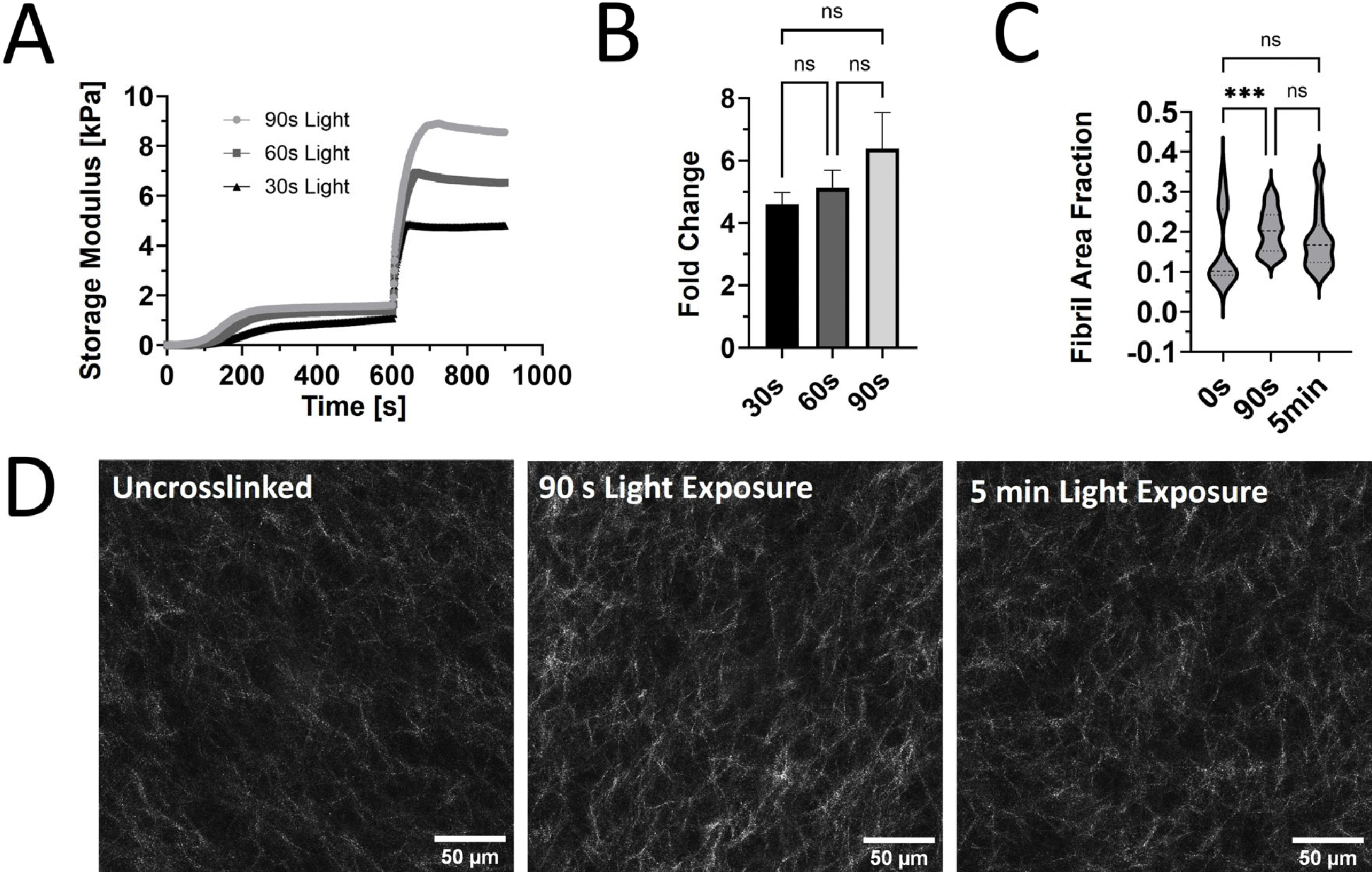
The viscoelastic response and microstructure of PhotoCol^®^ with Ru/SPS and burst light exposure. (**A**) Representative curves for the shear storage modulus (G’) versus time for PhotoCol^®^ with Ru/SPS with burst light exposure (405 nm; 30 s, 60 s, and 90 s) after 10 min of self-assembly. (**B**) Calculated fold change + SD before and after photo-crosslinking PhotoCol^®^ samples with Ru/SPS (ns = no significance). (**C**) The fibril area fraction. The threshold pixel area by total pixel area, was calculated via CellProfiler image analysis. Nonparametric data are displayed as violin plots (n = 3, n = 15, ns = no significance, and *** = *p* < 0.0001). (**D**) Confocal reflection microscopy images for uncrosslinked PhotoCol^®^ (0 s light exposure) and photo-crosslinked PhotoCol^®^ (90 s and 5 min. light exposure). White = collagen fibrils in reflectance; scale bar = 50 μm.

Our prior rheological tests used constant light exposure to ensure that the maximum stiffness was reached for each sample. However, burst light exposures of 30, 60, and 90 s after the initial self-assembly were used to assess the tunability of G’ for PhotoCol^®^ with Ru/SPS (Figure 2A). A dose-dependent trend was observed with increased maximum G’ values with increasing exposure time. However, when we calculated the fold change differences at each light exposure duration, increases in stiffness and dynamic range were not significantly different (Figure 2B). We expected to see significant increases in stiffness with increased exposure time, because a previous study that characterized methacrylated collagen and IRG observed significant differences [16]. However, since that study used 3.75 mg/mL methacrylated collagen versus 7.122 mg/mL PhotoCol^®^ in the current study, our higher working concentration might have contributed to the discrepancy. We chose a high working concentration to maximize overall stiffness values to approximate fibrotic tissue stiffness levels, but other approaches can be taken to generate a more tunable material. Visual assessment of the collagen fibril microstructure (confocal reflectance microscopy) of uncrosslinked PhotoCol^®^ with Ru/SPS compared to samples with 90 s and 5 min of photo-crosslinking showed minimal qualitative differences in fibril arrangement and uniformity (Figure 2D). All samples appeared to have homogenous fibril distribution with slight increases in fibril bundling and density in the photo-crosslinked samples. Quantitative analysis of fibril microstructure was performed using CellProfiler^™^ to calculate pixel density and obtain the fibril area fraction as an indicator of overall fibril density (Figure 2C). PhotoCol^®^ samples exposed to 30 s of light had a significantly higher fibril area fraction when compared to uncrosslinked samples, indicating an increase in density likely due to crosslinking. Samples exposed to 90 s of light were not significantly different from uncrosslinked or 30 s samples, which was unexpected given the visual similarities between 30 s and 90 s samples. However, the broad distribution of fibril area fractions for uncrosslinked samples likely contributed to the lack of significance and suggests a higher degree of variability in uncrosslinked samples.

### 2.3. PhotoCol^®^ Permeability Decreases with Photo-Crosslinking for 40 kDa Dextran

**Figure 3.**
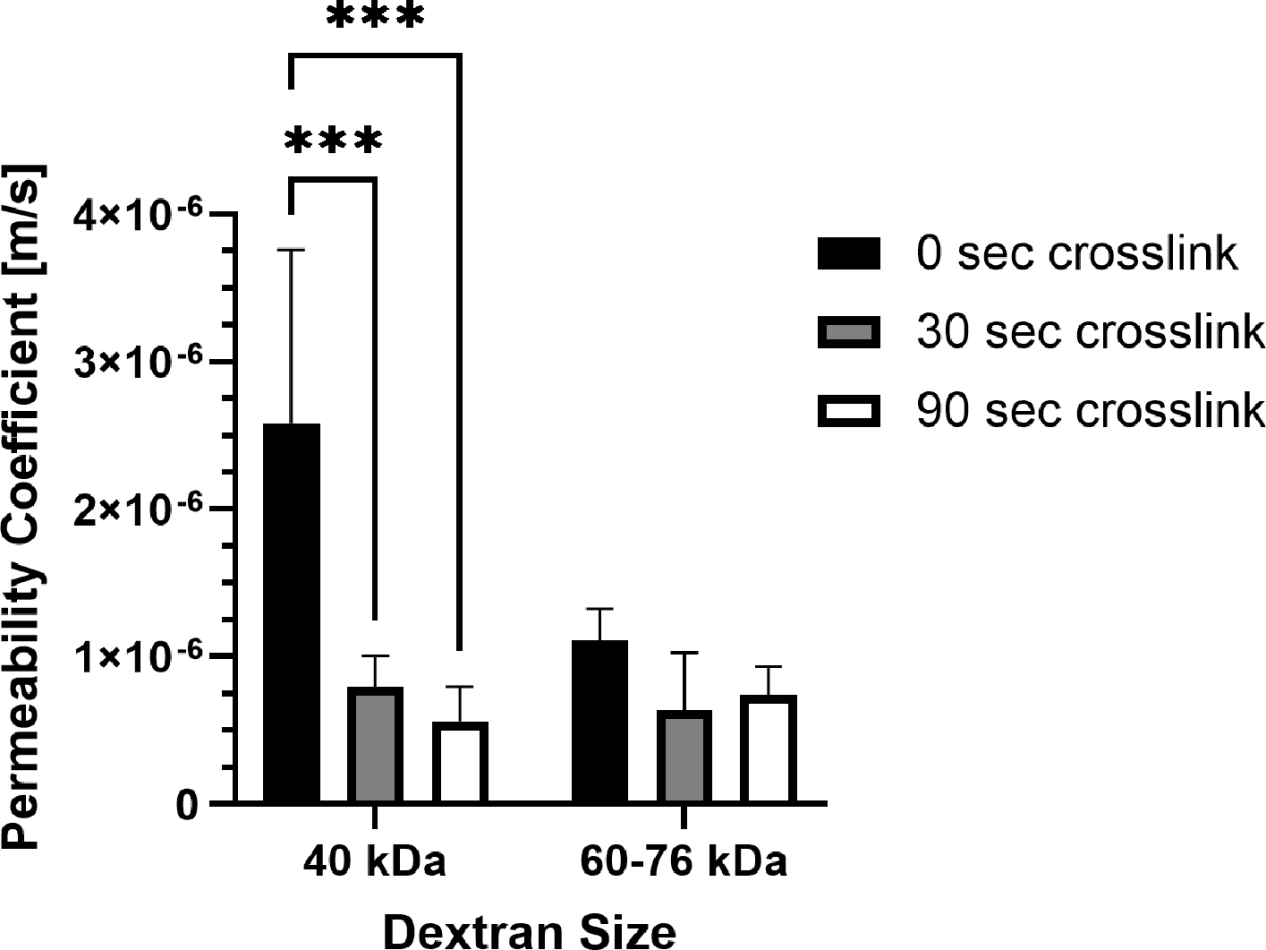
Permeability coefficients of PhotoCol^®^ with fluorescein isothiocyanate (FITC)-labeled dextran. Permeability coefficients were measured for PhotoCol^®^ with Ru/SPS and burst light exposure (0 s, uncrosslinked; 30 s and 90 s, photo-crosslinked). Bars represent mean + SD. Significance was determined with ANOVA used to compare the differences between photo-crosslinking duration and dextran size, and an unpaired Student’s *t*-test was used to compare differences between the two dextran sizes at each light duration (*** *p* < 0.001, n = 3–4 per group).

It is important to consider how molecular transport is affected by photo-crosslinking in methacrylated collagen prepared at higher concentration and stiffness levels. Permeability was impacted by photo-crosslinking for one of the two dextran sizes tested, but the duration of photo-crosslinking did not have an affect (Figure 3). For 40 kDa fluorescein isothiocyanate (FITC)-labeled dextran, permeability coefficients were significantly lower for PhotoCol^®^ samples photo-crosslinked for 30 s (7.96 × 10^−7^ ± 2.12 × 10^−7^ m/s) and 90 s (5.59 × 10^−7^ ± 2.40 × 10^−7^ m/s) compared to uncrosslinked samples (2.58 × 10^−6^ ± 1.18 × 10^−6^ m/s). Conversely, there was no statistical significance for 60–76 kDa dextran for uncrosslinked groups (1.11 × 10^−6^ ± 2.14 × 10^−7^ m/s) and PhotoCol^®^ samples photo-crosslinked for 30 s (6.38 × 10^−7^ ± 3.91 × 10^−7^ m/s) and 90 s (7.40 × 10^−7^ ± 1.97 × 10^−7^ m/s). There was also no difference in permeability between dextran sizes at each photo-crosslinking level.

Overall, this result indicates that the transport of molecules around 40 kDa was impacted by the degree of crosslinking, whereas the movement of larger molecules was more likely to be impeded regardless of crosslinking. We also performed permeability studies with 10 kDa dextran molecules in photo-crosslinked samples, and the associated permeability coefficients were not significantly different between uncrosslinked samples and 90 s light exposure samples, suggesting that the transport of smaller molecules was not affected by crosslinking. Within the microenvironment surrounding lymphatic capillaries, HDLECs will be exposed to soluble factors of varied size. Therefore, these permeability results reinforce that it is not only important to have a substrate that does not impede molecular transport, but it is also important to consider how mass transport is regulated in fibrotic microenvironments in vitro and in vivo. To deepen understanding of the implications of this work for lymphatic vasculature, additional dextran sizes should be investigated, particularly between 40 and 60 kDa. Inflammatory molecules like tumor necrosis factor (TNF)-α fall into this size range and are known to impact lymphatic function and leukocyte trafficking. In addition, the use of pore size as a metric should be tested to gain greater insight into permeability properties to explain this relationship.

### 2.4. PhotoCol^®^ Stiffness Alters HDLEC Morphology and Cell-Cell Junctions

**Figure 4.**
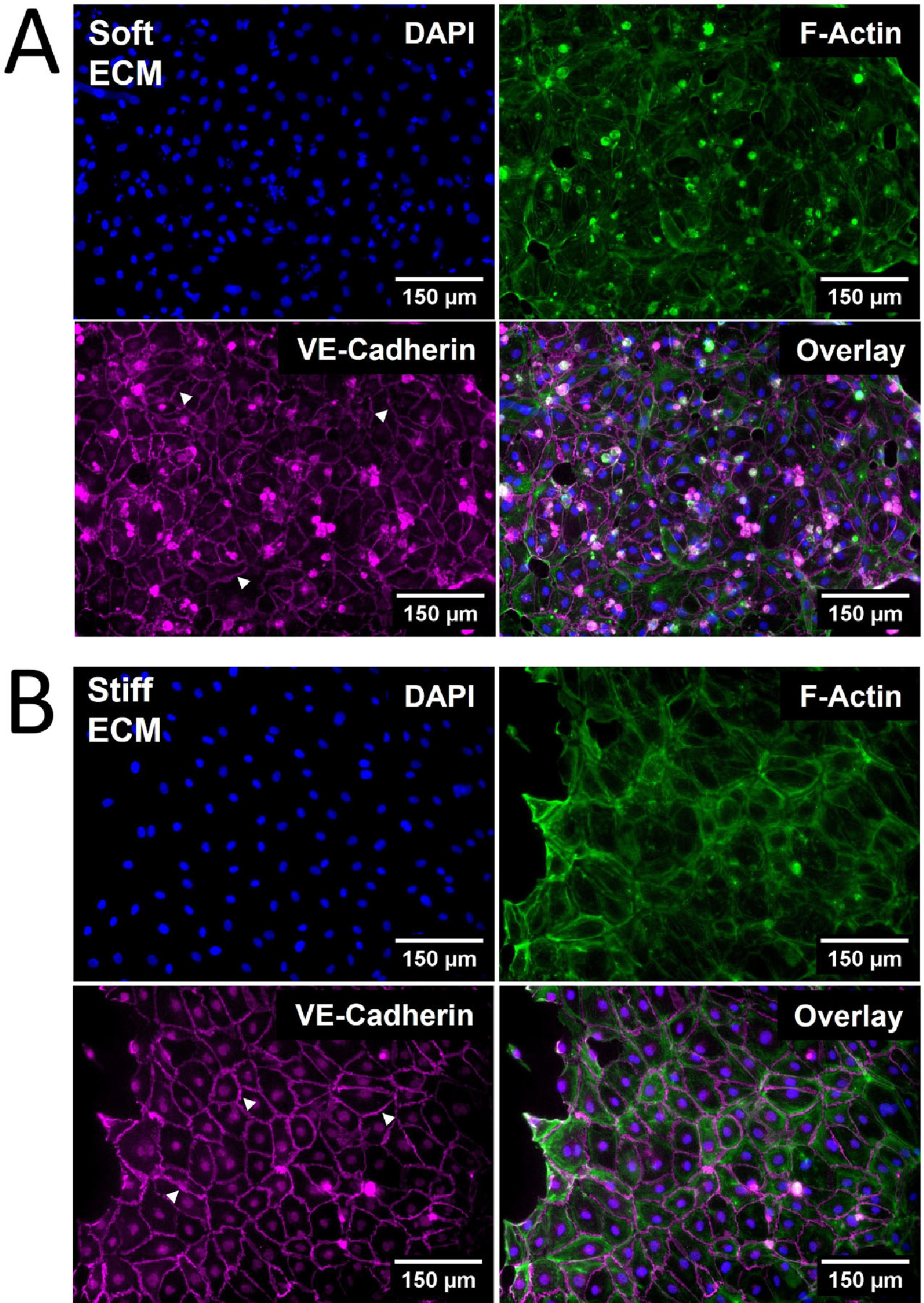
The qualitative morphological assessment of Human Dermal Lymphatic Endothelial Cells (HDLECs) on PhotoCol^®^. Representative images of HDLECs seeded on (**A**) uncrosslinked (soft, 0.5 kilopascal (kPa)) and (**B**) photo-crosslinked (90 s light exposure; stiff, 6 kPa) PhotoCol^®^ with Ru/SPS (fibronectin-coated). Samples were stained to visualize F-actin (green) and vascular endothelial (VE)-cadherin (purple) with 4′,6-diamidino-2-phenylindole (DAPI) nuclear counterstain (blue). White arrowheads point to cell–cell junctions used in quantitative analysis. Images were collected on a Keyence BZ-X series All-in-One Fluorescence Microscope (full focus z-stacks). Scale bar = 150 μm.

The viability of HDLECs on PhotoCol^®^ stiffened with Ru/SPS was confirmed via Live/Dead^™^ staining, allowing for stiffness response studies to be performed (see Supplementary Materials). Qualitative observations of HDLEC cultured on photo-crosslinked PhotoCol^®^ (stiff) showed larger cells with a greater cytoplasmic area and a more prominent VE-cadherin signal (Figure 4B) compared cells cultured on uncrosslinked PhotoCol^®^ (soft) (Figure 4A). Peripheral F-actin localization was also noted in HDLECs on photo-crosslinked gels, suggesting colocalization with VE-cadherin.

**Figure 5.**
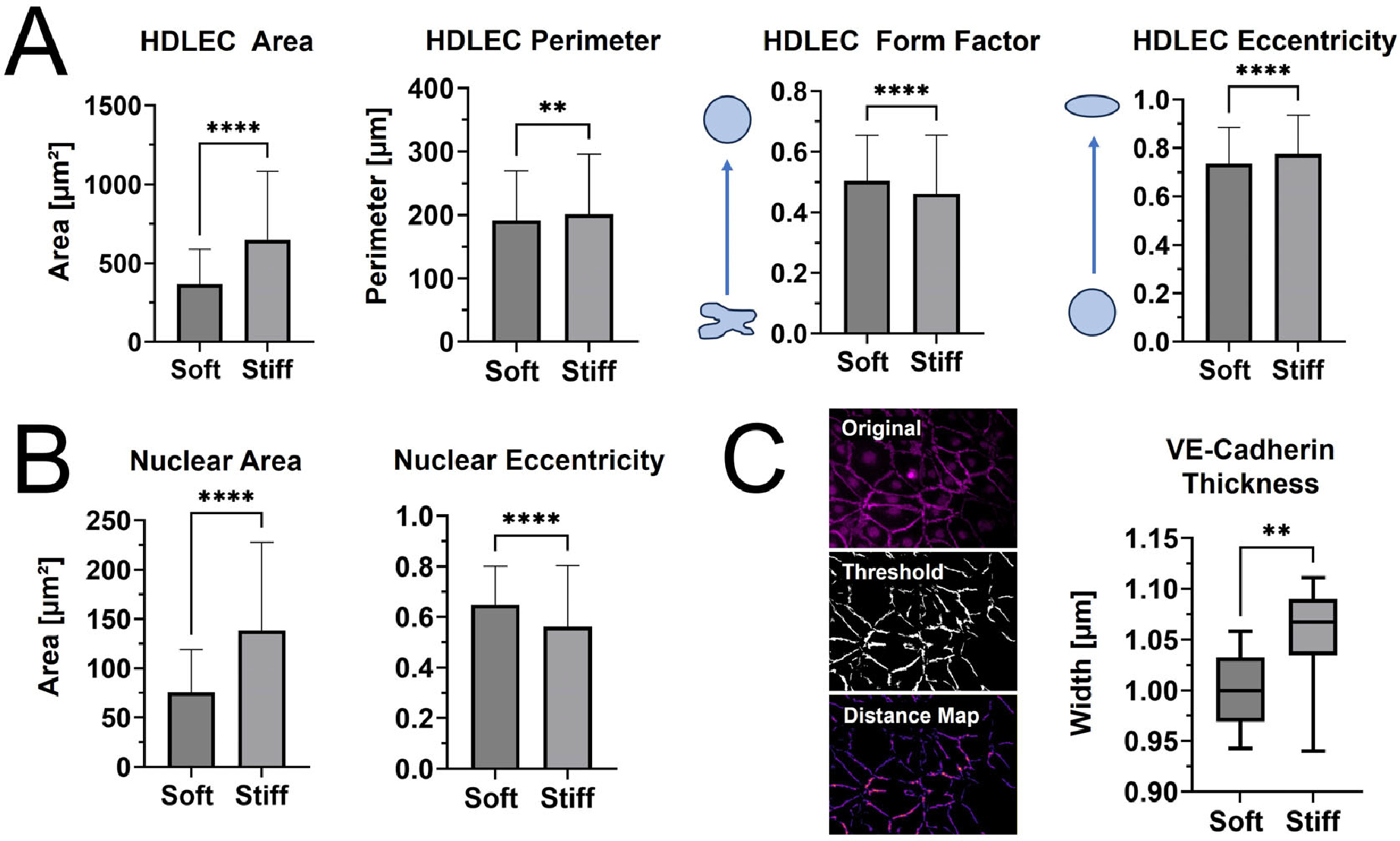
Qualitative morphological assessment of HDLECs on PhotoCol^®^. (**A**) Cell shape metrics (surface area, perimeter, form factor, and eccentricity) and (**B**) nuclear shape metrics (surface area and eccentricity for HDLECs on uncrosslinked (0.5 kPa) and photo-crosslinked (6 kPa) PhotoCol^®^) were calculated using CellProfiler^™^. Bars represent mean + SD. (**C**) VE-cadherin thickness (represented by width) for HDLECs on soft and stiff PhotoCol^®^. Images show steps to thickness quantification: original image, thresholded image (Cell Profiler^™^), and distance map for quantification (Vessel Analysis plug-in, ImageJ Version 2.15.1). Box and whisker plots represent minimum, maximum, and median widths across upper and lower quartiles (n = 300–700 cells across three images per group). Metrics were compared with Mann–Whitney U test (** *p* < 0.005 and **** *p* < 0.0001).

When cell and nuclear shape metrics and VE-cadherin thickness were measured in CellProfiler^™^ and FIJI, differences in morphology were confirmed (Figure 5). The average cellular and nuclear area significantly increased when seeded on stiffer PhotoCol^®^, while the increase in cell perimeter—an indicator of cell shape irregularity—was significant, yet moderate. Moreover, the decreased form factor observed in HDLECs on stiffer substrates also signaled more irregularly shaped cells, which aligns with the characteristic “oak leaf” shape that is used to describe LECs [20,21]. The perimeter result is also related to the slight increase in cell eccentricity—an elongation measure—on stiffer substrates because more circular shapes (i.e., lower eccentricity) have a lower perimeter/area ratio (Figure 5A). More elongated HDLECs may indicate a migratory and vessel forming morphology [7]. Conversely, even though there was an increase in nuclear area with stiffness, there was a decrease in eccentricity, suggesting more circular nuclei on stiffer substrates (Figure 5B).

HDLECs cultured on photo-crosslinked PhotoCol^®^ also showed thicker VE-cadherin junctions between cells (Figure 5C). The digital removal of dead and/or rounded cells or cell nuclei expressing a high VE-cadherin signal was performed to ensure that thickness measurements were not skewed and only captured junctions at the cell boundary (Figure 5C, representative images for “Original” vs. “Threshold”). Thicker VE-cadherin suggested increased zippering of the cell–cell junctions, with thinner VE-cadherin being closer to discontinuous button junctions. Button-like junctions represent a healthy junctional phenotype, whereas zippered junctions are often present in fibrotic environments [21]. The spread of VE-cadherin thickness values for HDLECs in the photo-crosslinked PhotoCol^®^ group overlaps with the uncrosslinked result. We can likely attribute the overlap to artifacts produced in the thresholding process that the software automatically included into the thickness measurements (i.e., numerous small, thin lines not associated with VE-cadherin). To remain consistent, the thickness measurement process was kept consistent across groups, and thus the artifacts were not removed.

## 3. Discussion

Recently, there has been an increased focus on the relationship between tissues stiffness and lymphatic vessel response, primarily with lymphatic capillaries and LECs [6–8]. For our study, we focused on tissue stiffness levels that are found in fibrotic disease conditions, since tissue stiffening from excess ECM deposition and crosslinking are major features of fibrosis that alter the biophysical and biochemical microenvironments surrounding lymphatic vasculature. Methacrylated type I collagen, combined with photoinitiators (LAP, IRG, or Ru/SPS) and light exposure (356–405 nm), was utilized as an “on-demand” stiffened material for fibrotic tissue disease modeling. The three photoinitiators were evaluated on how they altered the stiffening profiles of PhotoCol^®^, and Ru/SPS showed the highest average maximum stiffness, highest fold change after photo-crosslinking (i.e., dynamic range), and the quickest time to stiffness plateau. Importantly, the maximum stiffness of Ru/SPS PhotoCol^®^ reached an average of approximately 6 kPa, which is within the stiffness range of some diseased fibrotic tissue [22,23]. When Ru/SPS PhotoCol^®^ was tested for stiffness tunability using burst light exposures, a range of 5–9 kPa was achieved, but the fold change in the burst exposure was not statistically different despite an observable trend in increasing stiffness with light exposure. There was also a visual difference in how the PhotoCol^®^ fibrils formed under different stiffening conditions with longer, more densely packed fibrils with increased photo-crosslinking. The permeability of 40 kDa dextran was statistically different for photo-crosslinked versus uncrosslinked samples, but these effects were not significant for 60–76 kDa dextran. This result shows how molecular transport through methacrylated type I collagen is impacted by photo-crosslinking, which relates to how fibrosis-induced changes in tissues (i.e., crosslinking and stiffening) alters soluble factor signals that direct cell response. HDLEC morphology was also significantly affected by substrate stiffness with increased cell area and irregularity observed on stiffer PhotoCol©. These results were similar to other studies where soft substrates induced smaller cells, and stiff substrates induced flattened and spread-out morphologies [7]. Notably, our study also quantified the thickness differences in VE-cadherin cell junctions, which are known to be related to healthy and diseased LEC states [21]. Our approach extends the analysis of LEC response to include cell and nuclear shape metrics, along with VE-cadherin thickness. This morphological assessment is more robust and helpful in assessing LEC cultures, because morphology is less commonly used to describe changes in lymphatic vasculature outside of flow-based studies that focus on changes in elongation and alignment [24–26].

The use of methacrylated type I collagen in our study offers an alternative to unmodified type I collagen hydrogels that are unable to achieve high stiffness values. It can support 2D monolayer cultures of LECs, as well as LEC sprouting or outgrowth cultures within 3D. Methacrylated type I collagen can also reach stiffness levels that go beyond that of developing tissues, which shifts the potential for performing in vitro lymphatic studies at stiffness levels observed in diseased, fibrotic tissues. For instance, in conditions like pancreatic cancer with intratumoral and peritumoral lymphatic capillaries, tissue stiffness has been measured at 5.46 ± 3.18 kPa compared to 1.06 ± 0.25 kPa for normal pancreatic tissue [27]. The ability for methacrylated type I collagen to reach stiffness levels as high as 8 kPa positions us well to study lymphatics across a wide dynamic range of stiffnesses [16,17]. Methacrylated type I collagen has an additional advantage over other methacrylated materials, such as gelatin, HA, and various polysaccharides (e.g., dextran, alginate, chitosan) that are routinely used for cell and tissue culture. Although these materials can achieve high stiffness, they lack fibrillar structures on their own [28,29], which is important for recreating in vivo connective tissues and dictating ECM biophysical properties—mechanics, microstructure, and transport—that influence cell responses.

Photo-crosslinked methacrylated collagens from human, bovine, and rat tissue sources with LAP and IRG photoinitiators have high mechanical integrity and low cytotoxicity [16–19]. While stiffness and lack of toxicity are important to establish, there has been little work exploring other characteristics that impact cell behavior (e.g., permeability, and diffusivity). Our current work helps to fill that gap by evaluating permeability in PhotoCol^®^ with Ru/SPS. Prior studies have been performed to measure transport of soluble factors like dextran and bovine serum albumin through non-methacrylated type I collagens. Hsu et al. showed an inverse relationship between permeability speed and size of soluble factors but not within the context of changing collagen concentration or crosslinking [30]. Chen et al. investigated the interplay between concentration and crosslinking to establish release characteristics of methacrylated collagen hydrogels photo-crosslinked with IRG [19]. Using 70 kDa FITC-labeled dextran, diffusion was observed to be more dependent on collagen concentration rather than photo-crosslinking, with the highest diffusion rate observed in uncrosslinked collagen at a low concentration. They cited increased fibril density from a higher collagen concentration as the driving factor in hindering diffusion rather than increased stiffness from photo-crosslinking (~40 Pa vs. 200 Pa). However, the effects of crosslinking on fibril density were not explicitly addressed. High fibril density in our samples, resulting from a high collagen concentration, also appeared to hinder transport of higher molecular weight dextran (>60 kDa) compared to 40 kDa (Figure 3) and smaller dextran molecules. Yet, our results also showed a significant decrease in transport with increased photo-crosslinking, which might relate, in part, to the microstructural differences we observed in the fibril area fraction for uncrosslinked PhotoCol^®^ compared to samples with 30 s light exposure (Figure 2C). Although we did not observe significant differences in fibril area fraction between 30 s and 90 s light exposure samples or uncrosslinked and 90 s light exposure samples, the differences in permeability observed in the latter comparison supports the idea that the two samples have different microstructures. Collectively, these studies, including our study, provide insight into molecular transport through native and methacrylated collagen gels. We contribute to this area of study by widening the range of molecular sizes and matrix properties (e.g., stiffness and microstructure) to include more features that reflect fibrotic tissues.

Our results also align with other lymphatic studies showing show that HDLECs are responsive to differences in stiffness of the underlying substrate. Alderfer et al. [8] used modified HA/polyethylene glycol diacrylate (PEG-DA) composite hydrogels to tune ECM stiffness from 30–900 Pa and observed upregulation of genes involved in cell migration and tube formation (i.e., metalloproteinases 2 and 14) on softer matrices. This study also focused on the role of ECM stiffness in activating vascular endothelial growth factor receptor (VEGFR)-3, the primary receptor for vascular endothelial growth factor (VEGF)-C. On softer matrices, VEGFR-3 activation increased, which enabled more VEGF-C binding and subsequent formation of more extensive cord-like structures when compared to stiffer matrices. Frye et al. [7] cultured HDLECs on Softwell^™^ or Softslip^™^ dishes (Matrigen) to represent the tissue surrounding the cardinal vein (0.2 kPa,) and stiffer tissues throughout the body (4 kPa, embryonic cardinal vein; 8 and 12 kPa, muscle; 25 kPa, bone). Similar to Alderfer et al. [8], they observed upregulation of genes for cell–matrix adhesion, cell migration, and new lymphatic vessel growth (e.g., metalloproteinases 1, 2, and 10), as well as valve formation (e.g., GATA binding protein 2), while genes for cell proliferation were downregulated. These results were consistent with cells preparing for vessel formation and sprouting. In a concurrent in vivo study, LECs that migrated outside of the cardinal vein into a softer ECM were more elongated and spindle-shaped as a precursor to network formation, whereas LECs within the stiffer cardinal vein were flatter and appeared to be tightly attached to the underlying basement membrane [7]. Our quantitative assessment of LEC morphology was similarly aligned with HDLECs on soft, uncrosslinked PhotoCol^®^ being smaller (lower area) compared to HDLECs on stiff PhotoCol^®^ that had a higher cell area (flattened cells) with slightly more elongation (Figure 5). This result provides more insight into how LECs change morphologically in response to stiffness changes. In general, LEC responsiveness to ECM stiffness is expected since LECs within lymphatic capillaries are directly attached to the underlying ECM [13–15]. Interactions between LECs and collagen within connective tissues are uniquely tied to collagen structure. For instance, results from HDLEC studies in vitro and in vivo have shown stiffness-mediated changes in expression of matrix metalloproteinases that target a1(I) and a2(I) peptide chains within the type I collagen triple helix (e.g., metalloproteinases -1, -2, and -14) during cell migration and lymphatic sprouting [7–9,31]. These studies do not exclusively use type I collagen as a substrate for LECs, but the involvement of collagen-associated metalloproteinases shows the importance of leveraging collagen in lymphatic studies with and without stiffness changes. Moreover, we contribute to the field by using methacrylated type I collagen (PhotoCol^®^) at higher stiffness levels that are more representative of tissues undergoing fibrosis rather than development.

As lymphatic studies, including our own work, continue to move toward more mechanistic studies in the future, we can leverage our quantitative morphological assessments of cell shape and VE-cadherin junctions to relate alterations in mechanosensing pathways to morphological changes for more robust characterization of model systems. For instance, the Yes-associated protein (YAP)/transcriptional co-activator with PDZ binding motif (TAZ) pathway is notable for its mechanosensing capability of LECs when exposed to oscillatory shear and varied ECM stiffness [7,8,32]. Increased substrate stiffness has been shown to promote YAP/TAZ expression and translocation to the nucleus, which blocks Prox-1 and its downstream targets, VEFGR-3, and matrix metalloproteinase-14. Without robust activation of those two targets, lymphatic sprouting and subsequent formation of cord-like structures decreases. However, studies have not investigated whether this signaling pathway has any impact on cell morphology. Less is also known about relationships between VE-cadherin and either YAP/TAZ or ECM stiffness. Almost all lymphatic studies stain VE-cadherin, many assess VE-cadherin expression via western blot, but very few studies measure VE-cadherin morphology as we did in the current study [33]. Moreover, the driving factors behind VE-cadherin expression are not typically investigated. In our study, we not only observed thicker VE-cadherin junctions formed in HDLECs on stiff photo-crosslinked samples, but we also noted a potential association between VE-cadherin junctions and F-actin formation. A similar observation was noted in HDLECs on viscoelastic substrates [11]. Although neither study investigated the link between the two molecules within the context of HDLECs and stiffness, there are established connections between the VE-cadherin/catenin complex with actin [34]. These molecules are integrated through the processes of junction formation and maturation and remain associated during junction remodeling and maintaining junction integrity. Moreover, additional molecules, such as the Actin Related Protein (ARP)2/3 complex, α-catenin, and p120^ctn^, also help coordinate interactions. It will be important to investigate these mechanisms further within the context of lymphatic vasculature and ECM stiffness. Materials like methacrylated collagen will be helpful in these pursuits, because they retain collagen fibrillar microstructure that is important for cell attachment and appropriate force balance between cells and the surrounding ECM.

Through photo-crosslinking, we produced elastic hydrogels in the current studies that have covalent crosslinks that achieve static stiffness. However, there are temporal elements to the fibrotic process as excess ECM is deposited and crosslinked over time. Beyond stiffness, the biochemical environment also shifts with changes in ECM permeability that affect the movement of soluble factors and altered biodegradation that impacts tissue remodeling. Moreover, temporal changes in tissue stiffness and remodeling also affect ECM viscoelasticity. Fan et al. recently looked beyond static ECM stiffness and identified viscoelasticity as an important ECM property that impacts lymphatic morphogenesis and tube formation [11]. They combined supramolecular and covalent crosslinking to create dynamic HA hydrogels with tunable viscoelasticity that is spatially controlled with UV light exposure. Although the hydrogels exhibit the same maximum shear storage modulus (~1500 Pa) before and after UV irradiation, they differ in stress-relaxation behavior from static stiffness hydrogels (fully covalently crosslinked and elastic). In standard and photo-patterned viscoelastic HA substrates, HDLECs showed evidence of a higher degree of cell spreading and migration with increased F-actin stress fiber formation and focal adhesion assembly. We also observed greater cell area and F-actin formation on our photo-crosslinked samples, even though our stiffness levels were ~4 times higher than Fan et al. HDLECs on viscoelastic HA hydrogels also formed a more extensive and branched lymphatic tube network compared to elastic (static) hydrogels with increased expression of characteristic lymphatic markers such as lymphatic vessel endothelial hyaluronan receptor-1 (LYVE-1), Prospero homeobox protein 1 (Prox-1), podoplanin, and VEGFR-3. Matrix metalloproteinases 1, 2, and 14, which are involved in ECM degradation and remodeling events necessary for tube formation, also increased on viscoelastic hydrogels. Interestingly, these are some of the same markers that others observed as being expressed at higher levels on softer substrates (30–200 Pa) [7,8]. Collectively, these observations and study results provide rationale for why viscoelasticity and temporal stiffening are important factors to consider beyond static stiffness when modeling dynamic processes like fibrosis. These differences may also be a contributing factor to why in vitro lymphatic capillaries do not match the dense, non-functional capillary beds seen in vivo in disease.

Even though the current work focuses on a new material approach to investigate the lymphatic response to ECM stiffness, we recognize other work within the field that is making progress toward improved understanding of the lymphatic system. Beyond our work and other work with natural materials that were previously discussed, Hooks et al. recently used a synthetic poly(ethylene glycol) (PEG) hydrogel functionalized with four maleimide groups (PEG-4MAL) and binding arginylglycylaspartic acid (RGD) ligands to observe the relationship between matrix elasticity (stiffness), ligand binding density, and degradability on lymphatic sprouting [35]. Their study was designed to target one of the drawbacks of collagen, being that ligand density and stiffness profiles of gels are not independent when using collagen concentration to alter collagen stiffness. They were able to control matrix elasticity via PEG weight percentage without altering the RGD ligand density and generate matrices at 680 Pa with comparable ligand density to 2 mg/mL collagen at 20 Pa. Moreover, they showed successful lymphatic sprouting in vitro and functional grafting into host vasculature in vivo. Although they were able maintain ligand density at increased stiffness levels, methacrylated collagen behaves similarly by achieving multiple stiffness levels via photo-crosslinking at a single collagen concentration [16]. Methacrylated collagens are also capable of being integrated into lymphatic-on-a-chip models, which are popular models for studying lymphatic sprouting and growth [25,36–40]. Lymphatic capillaries sprout into ECM materials from LEC-lined channels, usually guided by a gradient of growth factors such as VEGF-C and sphingosine 1 phosphate [24]. Disease is currently a part of lymphatic-on-a-chip modeling; however, the focus tends to be on using soluble factors and co-cultures to recreate a disease environment. As with most lymphatic capillary models, ECM-based features of fibrosis have not been included in lymphatic-on-a-chip models. Therefore, there is an opportunity to merge the two approaches to advance the field of lymphatic modeling to include more robust disease models with altered stiffness and transport properties that impact LEC behavior.

## 4. Materials and Methods

Table 1 includes all product information for reagents, consumables, major equipment, and software used in the study, including catalog number and company.

### 4.1. Preparation of Methacrylated Collagen Hydrogels (PhotoCol^®^)

Lyophilized methacrylated type I collagen (PhotoCol^®^, Advanced BioMatrix, Carlsbad, CA, USA) was solubilized on a rotator at 4 °C in sterile 20 mM acetic acid to a final concentration of 8 mg/mL. Three photoinitiators were prepared according to manufacturer (Advanced BioMatrix) protocols: Lithium phenyl-2,4,6-trimethylbenzoylphosphinate (LAP, 17 mg/mL stock volume), Irgacure 2959 (IRG, 10% stock), and Ruthenium (Ru)/Sodium Persulfate (SPS) (Ru at 37.4 mg/mL and SPS at 119 mg/mL stock volume). A neutralization solution (Advanced BioMatrix) at 8% (*v*/*v*) (7.407 mg/mL) and the photoinitiator at 2% (*v*/*v*) were mixed with PhotoCol^®^ to make the final hydrogel mixture for each photoinitiator (LAP—7.262 mg/mL; IRG—7.334 mg/mL; and Ru/SPS—7.122 mg/mL). Neutralized PhotoCol^®^ hydrogels with the photoinitiator were incubated at 37 °C for a minimum of 30 min for hydrogel self-assembly prior to photo-crosslinking in subsequent studies.

### 4.2. Rheological Assessment of PhotoCol^®^ Viscoelasticity

Matrix viscoelastic properties (shear storage modulus, G′ and shear loss modulus, G″) were measured using a MCR 302e WESP rheometer (Anton Paar, Graaz, Austria) with a quartz stage to photo-crosslink PhotoCol^®^ hydrogel samples (365–405 nm) during testing. Frequency and strain sweeps were conducted in oscillatory shear on fully crosslinked and un-crosslinked hydrogels to determine the linear viscoelastic region. Temporal changes in stiffness in response to photo-crosslinking were conducted in time sweeps (constant: 0.1% strain, 1 Hz). Hydrogel samples were allowed to self-assemble for 10 min at 37 °C before exposure to one of two light exposure conditions—constant and burst exposure—using 365 or 405 nm UV mounted LED (Thorlabs, Newton, NJ, USA). Constant light was applied for 20 min after self-assembly for complete photo-crosslinking and maximum stiffness (G’). Burst light exposures consisted of a single light pulse for 30, 60, or 90 s after self-assembly with continued measurement for 10 min after light exposure.

Using MATLAB (MathWorks, Natick, MA, USA), a locally weighted regression was used on each replicate to create a fitted curve that could be analyzed [41]. The first derivate was taken of these regressions to determine rate of change in stiffness. A threshold of 3 Pa/s rate was used to determine the regions of plateau and regions of stiffening. Average stiffness values of the plateau regions before and after light exposure, within the regions of stiffening, were used to calculate fold change by the ratio of each replicate’s post-stiffening average to the same replicate’s pre-stiffening average. The post-stiffening averages of each replicate were then averaged to give each group’s average maximum stiffness. The average number of seconds that the rate of change in stiffening was above the 3 Pa/s threshold was used to calculate the amount of time to stiffness plateau.

### 4.3. Microstructural Assessment of PhotoCol^®^ Hydrogels

Confocal laser scanning microscopy in reflectance mode was used to obtain images of fibrillar collagen microstructure for qualitative assessment. All samples were prepared in Nunc^™^ Lab-Tek^™^ II Chambered Coverglass #1.5 slides (ThermoFisher Scientific, Waltham, MA, USA). Briefly, PhotoCol^®^ hydrogels with Ru/SPS were neutralized and allowed to self-assemble for 30 min at 37 °C as previously described. Photo-crosslinked samples were exposed to 405 nm light for 90 s (longest burst exposure) or 5 min (sufficient time to reach maximum G’) using a 405 nm UV mounted LED (Thorlabs). Images were collected in reflectance mode with a Leica TCS SP5 Spectral Confocal Microscope (Leica Microsystems, Wetzlar, Germany) using a 40× air objective (3D z-stack). Each z-stack began 20 μm above the cover glass and consisted of 15 slices with 2 μm spacing between slices (30 μm total thickness). Three stacks were taken per sample. Using CellProfiler^™^ (Broad Institute, Cambridge, MA, USA), images were enhanced and clarified with the “openlines” function. Threshold images were then created from these and the pixel area ratio was calculated to determine the fibril area ratio.

### 4.4. Permeability Assessment of PhotoCol^®^ Hydrogels

A transwell membrane insert system protocol adapted from Hsu et al. [30] was used to characterize the permeability of PhotoCol^®^ hydrogels with the Ru/SPS photoinitiator. Neutralized PhotoCol^®^ (60 μL; 7.122 mg/mL) was polymerized for 60 min at 37 °C on an 8.0 μm PET membrane 24-well transwell insert (Corning, New York, NY, USA) within a tissue-cultured treated 24-well plate (Corning). For photo-crosslinking, transwell inserts containing PhotoCol^®^ were removed and exposed to 405 nm near UV light for 30 or 90 s. Lyophilized human fibronectin (Advanced BioMatrix) was reconstituted in Milli-Q water and diluted in 1X PBS (Fisher Bioreagents, Waltham, MA, USA) to achieve 5 μg/cm^2^ (0.03 mg/mL) on the PhotoCol^®^ surface. Samples were then incubated at 37 °C for 1 h to establish a fibronectin coating and submerged in 1X PBS (overnight at 37 °C) to remove any excess Ru/SPS from the PhotoCol^®^ gels. A 2 mg/mL solution of fluorescein isothiocyanate (FITC)-labeled dextran at 40 kDa or 60–76 kDa (Sigma Aldrich, St. Louis, MO, USA) was added on top of the fibronectin-coated PhotoCol^®^ samples within the inserts as the donor solution, while 1X PBS was pipetted into the outer well to serve as the acceptor solution. Solution volumes were controlled to be at equal heights to minimize flow due to hydrostatic pressure. PhotoCol^®^ with FITC-dextran was incubated at 37 °C, and 20 μL was removed from the acceptor solution at 1 h increments for a total of 3–5 h. The sampled solution was then diluted at 1:25 in 1X PBS and transferred to a black-walled polystyrene 96-well plate (Corning) to be read on a PerkinElmer VICTOR Nivo plate reader (Revvity, Waltham, MA, USA) (480 nm excitation/530 nm emission). A standard curve (0–0.0125 mg/mL of FITC-dextran) was used to calculate the concentration of measured samples. To calculate the permeability coefficient, Fick’s second law was used, using the dimensions of the PhotoCol^®^ sample (i.e., surface area) and the change in concentration of the acceptor solution in the linear region of permeability.

### 4.5. Lymphatic Endothelial Cell Culture

Human Dermal Lymphatic Endothelial Cells (HDLECs) isolated from adult skin (PromoCell, Heidelberg, Germany) were maintained according to manufacturer’s instructions in endothelial growth media with MV2 growth supplements (PromoCell) that contain the following: fetal calf serum (5% *v*/*v*), epidermal growth factor (recombinant human, 5 ng/mL), basis fibroblast growth factor (recombinant human, 10 ng/mL), insulin-like growth factor (Long R3 IGF, recombinant human, 20 ng/mL), vascular endothelial growth factor 165 (recombinant human, 0.5 ng/mL), ascorbic acid (1 μg/mL), and hydrocortisone (0.2 μg/mL) (PromoCell). All cell culture surfaces were coated with human fibronectin (3.5 μg/cm^2^; Advanced BioMatrix) prior to HDLEC seeding to promote cell attachment. Cells were passaged and maintained in Fisherbrand^™^ Vented Cap Surface Treated Sterile Tissue Culture Flasks (Fisher Scientific, Waltham) until ready for experimental use. Cells were maintained at 37 °C in a humidified incubator (5% CO_2_) and passaged at 70–90% confluency. All cells were used between passages 6 and 12 for experiments.

### 4.6. Morphological Assessment of Lymphatic Endothelial Cell Stiffness Response

HDLEC viability was confirmed via direct and indirect exposure to Ru/SPS followed with a Live/Dead^™^ stain (Invitrogen, Waltham, MA, USA) and quantified using FIJI (See Supplementary Materials). HDLECs were characterized by their morphological response to hydrogels at different stiffness levels. Cells were seeded in a 15-well glass bottom μ-slide (ibidi GmbH, Gräfelfing, Germany) at 10,000 cells/cm^2^ on top of fibronectin-coated PhotoCol^®^ prepared with Ru/SPS (photo-crosslinking, 90 s of light exposure, and high G’) and without photoinitiator (uncrosslinked, no light exposure, and low G’) for a total culture time of 3 days (37 °C, 5% CO_2_). All groups were fixed with 4% paraformaldehyde (ThermoFisher Scientific) in 1X PBS for 15 min at 37 °C and permeabilized using 0.1% of Triton^™^ X-100 (Sigma Aldrich) diluted in 1X PBS. Samples were stained with Alexa Fluor^™^ 488 Phalloidin (1:400, ThermoFisher Scientific) to visualize F-actin and counterstained with 4′,6-diamidino-2-phenylindole (300 nM DAPI; ThermoFisher Scientific) to visualize the nuclei. For immunostaining of characteristic HDLEC markers, fixed samples were blocked with bovine albumin fraction V (7.5% solution; ThermoFisher Scientific) diluted in 1X PBS to 1% and incubated with primary antibodies for VE-cadherin (1:1000 ThermoFisher Scientific). Samples were then rinsed and incubated with Alexa Fluor^™^ Plus 647-conjugated secondary antibody donkey anti-rabbit IgG (H + L) (1:200, ThermoFisher Scientific). All samples were imaged with a Keyence BZX810 All-in-One Fluorescence Microscope (KEYENCE Corp. of America, Itasca, IL, USA). Images were collected at 20× magnification for cellular and nuclear area, cellular and nuclear eccentricity, cellular form factor, and VE-cadherin thickness, utilizing the Keyence BZ-X800 analysis software (Version 1.1.2.4) to create full focus images from z-stacks. The threshold of these images was obtained utilizing CellProfiler^™^, removing signal from cell nuclei and noise (including rounded dead cells with prominent staining) before obtaining the threshold image of just the VE-cadherin cellular outline (see “Original” and “Threshold” images in Figure 5C to show signal removal). Primary (nuclei) and secondary objects (cell body) were obtained and quantified using CellProfiler^™^ as well. Threshold images were then quantified in FIJI, using distance map and Vessel Analysis plugins. Due to the visual similarity between threshold images of vasculature and threshold images of cell boundaries, the Vessel Analysis plugin was able to accurately obtain average vessel diameters of four random regions of interest in each image (n = 4, n = 3).

### 4.7. Statistical Analysis

Statistical analysis was carried out using GraphPad Prism 10.0 (GraphPad Software Inc., La Jolla, CA, USA). Stiffness data were expressed as mean storage modulus + standard deviation (SD) without removal of outliers. Permeability data were expressed as a mean + SD after outliers were removed using Grubbs’ test (α = 0.05). Morphological values were expressed as mean ± SD without removal of outliers. Normality was verified through a Shapiro–Wilk normality test using α = 0.05. Multiple comparisons were performed using one-way analysis of variance (ANOVA) (maximum stiffness, fold change, and fibril area fraction) or a two-way ANOVA (permeability), and parametric data were analyzed using Tukey’s post-hoc method (significance: * *p* < 0.05, ** *p* < 0.001, *** *p* < 0.0001, and **** *p* < 0.00001). Nonparametric data for morphological analysis were analyzed with the Mann–Whitney U test (significance: (** *p* < 0.005 and **** *p* < 0.0001).

## 5. Conclusions

The progressive nature of fibrosis includes dynamic changes in the biophysical and biochemical cues provided to the affected cells. Lymphatic vascular growth and function is heavily affected during the progression of fibrosis and the related stiffening of the surrounding tissue. To prepare an in vitro model that best recapitulates fibrotic stiffening, we characterized the stiffening profiles of methacrylated type I collagen (PhotoCol^®^) with photo-crosslinking and identified Ru/SPS as the photoinitiator that produced the highest maximum storage modulus and greatest dynamic range of stiffness values that approximate normal to fibrotic tissue stiffness. As others have found, we confirmed that HDLECs are responsive to changes in ECM stiffness. The observed differences in cellular and nuclear area and irregularity, combined with VE-cadherin thickness measurements, show elements of LEC maturation that relate to growth and function. Moreover, they offer more robust morphological characterization for HDLECs that we and others can leverage in future mechanism-based studies to understand specific drivers of stiffness-induced LEC changes. Overall, our focus on considerably higher ECM stiffness values places our work within the disease modeling space rather than lymphatic development. Our findings also establish methacrylated type I collagen as a tunable, natural material that demonstrates the capability to be used for lymphatic disease modeling that will improve our understanding of the biophysical and biochemical effects of fibrosis on lymphatic vasculature.

## Supplementary Material

supplementary material

## Figures and Tables

**Table 1.** Materials, cells, software, catalog number, and company.

Materials (Cells and Cell Culture)	Catalog Number	Company
Human Dermal Lymphatic Endothelial Cells (HDLECs), Adult, Cyropreserved	#C-12217	PromoCell
Endothelial Cell Growth Medium MV2 Kit With Supplement Pack	#C-22121	PromoCell
Materials (Reagents and Antibodies)	Catalog Number	Company
PhotoCol^®^ Methacrylated Collagen	#5198-100MG	Advanced BioMatrix
Lithium phenyl-2,4,6-trimethylbenzoylphosphinate (405 nm)	#5269	Advanced BioMatrix
Irgacure 2959 (365 nm)	#5200	Advanced BioMatrix
Ruthenium/Sodium Persulfate (400–450 nm)	#5248	Advanced BioMatrix
Fibronectin, Lyophilized (Human)	#5080	Advanced BioMatrix
Phosphate Buffered Saline, 10X Solution	#BP3991	Fisher Bioreagents
Fluorescein isothiocyanate–dextran, 40 kDa	#FD40S-250MG	Sigma Aldrich
Fluorescein isothiocyanate–dextran, 60–76 kDa	#FD70S-250MG	Sigma Aldrich
Pierce^™^ 16% Formaldehyde (*w*/*v*), Methanol-free	#28908	ThermoFisher Scientific
Triton^™^ X-100	#T8787-50ML	Sigma Aldrich
Alexa Fluor^™^ 488 Phalloidin	#A12379	ThermoFisher Scientific
4’,6-diamidino-2-phenylindole, dihydrochloride (DAPI)	#62247	ThermoFisher Scientific
Bovine Albumin Fraction V (7.5% solution)	#15260037	ThermoFisher Scientific
VE-cadherin Polyclonal Antibody (Rabbit Anti-Human)	#PA5-19612	ThermoFisher Scientific
Donkey anti-Rabbit IgG (H + L) Highly Cross-Adsorbed Secondary Antibody, Alexa Fluor^™^ Plus 647	# A32795	ThermoFisher Scientific
Materials (Consumables)	Catalog Number	Company
Nunc^™^ Lab-Tek^™^ II Chambered Coverglass	#155360	ThermoFisher Scientific
Costar^®^ 24-well Clear TC-treated Multiple Well Plates, Individually Wrapped, Sterile	#3524	Corning
Falcon^®^ Permeable Support for 24-well Plate with 8.0 μm Transparent PET Membrane, Sterile	#393097	Corning
Corning^®^ 96-well Flat Clear Bottom Black Polystyrene TC-treated Microplates, Individually Wrapped, with Lid, Sterile	#3603	Corning
μ-Slide 15 Well, 3D Glass Bottom	#81507	ibidi GmbH
Fisherbrand^™^ Surface Treated Sterile Tissue Culture Flasks, Vented Cap	#FB012937	Fisher Scientific
Materials (Equipment and Software)	Catalog/Model Number	Company
MCR 302e WESP rheometer	#241353	Anton Paar
UV Mounted LED	#M365LP1	Thorlabs
UV Mounted LED	#M405LP1	Thorlabs
Leica TCS SP5 Spectral Confocal Microscope	--	Leica Microsystems
Victor Nivo 6T Multimode Plate Reader	#HH35000500	Revvity Health Sciences
Keyence All-in-One Fluorescence Microscope	#BZ-X810	KEYENCE Corp. of America
GraphPad Prism 10.0	--	GraphPad Software Inc.

## Data Availability

Raw data supporting these conclusions will be available on request from the authors.

## References

[1] Petrova TV; Koh GY Organ-specific lymphatic vasculature: From development to pathophysiology. J. Exp. Med. 2018, 215, 35–49. 29242199 10.1084/jem.20171868 PMC5748863

[2] Avraham T; Daluvoy S; Zampell J; Yan A; Haviv YS; Rockson SG; Mehrara BJ Blockade of transforming growth factor-β1 accelerates lymphatic regeneration during wound repair. Am. J. Pathol. 2010, 177, 3202–3214. 21056998 10.2353/ajpath.2010.100594 PMC2993295

[3] Avraham T; Zampell JC; Yan A; Elhadad S; Weitman ES; Rockson SG; Bromberg J; Mehrara BJ Th2 differentiation is necessary for soft tissue fibrosis and lymphatic dysfunction resulting from lymphedema. FASEB J. 2013, 27, 1114–1126. 23193171 10.1096/fj.12-222695 PMC3574290

[4] Ogata F; Fujiu K; Matsumoto S; Nakayama Y; Shibata M; Oike Y; Koshima I; Watabe T; Nagai R; Manabe I Excess Lymphangiogenesis Cooperatively Induced by Macrophages and CD4+ T Cells Drives the Pathogenesis of Lymphedema. J. Investig. Dermatol. 2016, 136, 706–714. 27015456 10.1016/j.jid.2015.12.001

[5] Avraham T; Yan A; Zampell JC; Daluvoy SV; Haimovitz-Friedman A; Cordeiro AP; Mehrara BJ Radiation therapy causes loss of dermal lymphatic vessels and interferes with lymphatic function by TGF-β1-mediated tissue fibrosis. Am. J. Physiol.-Cell Physiol. 2010, 299, 589–605. 10.1152/ajpcell.00535.2009 PMC2944320 20519446

[6] Ruliffson BNK; Whittington CF Regulating Lymphatic Vasculature in Fibrosis: Understanding the Biology to Improve the Modeling. Adv Biol. 2023, 7, e2200158. 10.1002/adbi.202200158 36792967

[7] Frye M; Taddei A; Dierkes C; Martinez-Corral I; Fielden M; Ortsäter H; Kazenwadel J; Calado DP; Ostergaard P; Salminen M; Matrix stiffness controls lymphatic vessel formation through regulation of a GATA2-dependent transcriptional program. Nat. Commun. 2018, 9, 1511. 29666442 10.1038/s41467-018-03959-6 PMC5904183

[8] Alderfer L; Russo E; Archilla A; Coe B; Hanjaya-Putra D Matrix stiffness primes lymphatic tube formation directed by vascular endothelial growth factor-C. FASEB J. 2021, 35, e21498. 33774872 10.1096/fj.202002426RR PMC8011948

[9] Detry B; Erpicum C; Paupert J; Blacher S; Maillard C; Bruyère F; Pendeville H; Remacle T; Lambert V; Balsat C; Matrix metalloproteinase-2 governs lymphatic vessel formation as an interstitial collagenase. Blood 2012, 119, 5048–5056. 22490679 10.1182/blood-2011-12-400267

[10] Saha S; Fan F; Alderfer L; Graham F; Hall E; Hanjaya-Putra D Synthetic hyaluronic acid coating preserves the phenotypes of lymphatic endothelial cells. Biomater. Sci. 2023, 11, 7346–7357. 37789798 10.1039/d3bm00873h PMC10628678

[11] Fan F; Su B; Kolodychak A; Ekwueme E; Alderfer L; Saha S; Webber MJ; Hanjaya-Putra D Hyaluronic Acid Hydrogels with Phototunable Supramolecular Cross-Linking for Spatially Controlled Lymphatic Tube Formation. Cite This ACS Appl. Mater. Interfaces 2023, 15, 58195. 10.1021/acsami.3c12514 PMC10739586 38065571

[12] Pehrsson M; Mortensen JH; Manon-Jensen T; Bay-Jensen AC; Karsdal MA; Davies MJ Enzymatic cross-linking of collagens in organ fibrosis—resolution and assessment. Expert. Rev. Mol. Diagn. 2021, 21, 1049–1064. 34330194 10.1080/14737159.2021.1962711

[13] Danussi C; Spessotto P; Petrucco A; Wassermann B; Sabatelli P; Montesi M; Doliana R; Bressan GM; Colombatti A Emilin1 Deficiency Causes Structural and Functional Defects of Lymphatic Vasculature. Mol. Cell Biol. 2008, 28, 4026–4039. 18411305 10.1128/MCB.02062-07 PMC2423131

[14] Wiig H; Keskin D; Kalluri R Interaction between the extracellular matrix and lymphatics: Consequences for lymphangiogenesis and lymphatic function. Matrix Biol. 2010, 29, 645–656. 20727409 10.1016/j.matbio.2010.08.001 PMC3992865

[15] Chen J; Alexander JS; Orr AW Integrins and Their Extracellular Matrix Ligands in Lymphangiogenesis and Lymph Node Metastasis. Int. J. Cell Biol. 2012, 2012, 1–12. 10.1155/2012/853703 PMC3296286 22505936

[16] Gaudet ID; Shreiber DI Characterization of methacrylated Type-I collagen as a dynamic, photoactive hydrogel. Biointerphases 2012, 7, 25. 22589068 10.1007/s13758-012-0025-y PMC4243547

[17] Brinkman WT; Nagapudi K; Thomas BS; Chaikof EL Photo-Cross-Linking of Type I Collagen Gels in the Presence of Smooth Muscle Cells: Mechanical Properties, Cell Viability, and Function. Biomacromolecules 2003, 4, 890–895. 12857069 10.1021/bm0257412

[18] Ali SM; Patrawalla NY; Kajave NS; Brown AB; Kishore V Species-Based Differences in Mechanical Properties, Cytocompatibility, and Printability of Methacrylated Collagen Hydrogels. Biomacromolecules 2022, 23, 5137–5147. 36417692 10.1021/acs.biomac.2c00985 PMC11103796

[19] Chen CL; Wei SY; Chen WL; Hsu TL; Chen YC Reconstructing vascular networks promotes the repair of skeletal muscle following volumetric muscle loss by pre-vascularized tissue constructs. J. Tissue Eng. 2023, 14, 20417314231201231. 37744322 10.1177/20417314231201231 PMC10517612

[20] Baluk P; Fuxe J; Hashizume H; Romano T; Lashnits E; Butz S; Vestweber D; Corada M; Molendini C; Dejana E; Functionally specialized junctions between endothelial cells of lymphatic vessels. J. Exp. Med. 2007, 204, 2349–2362. 17846148 10.1084/jem.20062596 PMC2118470

[21] Baluk P; McDonald DM Buttons and Zippers: Endothelial Junctions in Lymphatic Vessels. Cold Spring Harb. Perspect. Med. 2022, 12, a041178. 35534209 10.1101/cshperspect.a041178 PMC9643678

[22] Ng MR; Brugge JS A Stiff Blow from the Stroma: Collagen Crosslinking Drives Tumor Progression. Cancer Cell 2009, 16, 455–457. 19962663 10.1016/j.ccr.2009.11.013

[23] Samani A; Zubovits J; Plewes D Elastic moduli of normal and pathological human breast tissues: An inversion-technique-based investigation of 169 samples. Phys. Med. Biol. 2007, 52, 1565–1576. 17327649 10.1088/0031-9155/52/6/002

[24] Wang K-C; Yeh Y-T; Nguyen P; Limqueco E; Lopez J; Thorossian S; Guan K-L; Li Y-SJ; Chien S Flow-dependent YAP/TAZ activities regulate endothelial phenotypes and atherosclerosis. Proc. Natl. Acad. Sci. USA 2016, 113, 11525–11530. 27671657 10.1073/pnas.1613121113 PMC5068257

[25] Akbari E; Spychalski GB; Rangharajan KK; Prakash S; Song JW Flow dynamics control endothelial permeability in a microfluidic vessel bifurcation model. Lab Chip 2018, 18, 1084–1093. 29488533 10.1039/c8lc00130h PMC7337251

[26] Choi D; Park E; Jung E; Seong YJ; Hong M; Lee S; Burford J; Gyarmati G; Peti-Peterdi J; Srikanth S; ORAI1 Activates Proliferation of Lymphatic Endothelial Cells in Response to Laminar Flow Through Krüppel-Like Factors 2 and 4. Circ. Res. 2017, 120, 1426–1439. 28167653 10.1161/CIRCRESAHA.116.309548 PMC6300148

[27] Rubiano A; Delitto D; Han S; Gerber M; Galitz C; Trevino J; Thomas RM; Hughes SJ; Simmons CS Viscoelastic properties of human pancreatic tumors and in vitro constructs to mimic mechanical properties. Acta Biomater. 2018, 67, 331–340. 29191507 10.1016/j.actbio.2017.11.037 PMC5797706

[28] Tabatabaei F; Moharamzadeh K; Tayebi L Fibroblast encapsulation in gelatin methacryloyl (GelMA) versus collagen hydrogel as substrates for oral mucosa tissue engineering. J. Oral. Biol. Craniofacial Res. 2020, 10, 573–577. 10.1016/j.jobcr.2020.08.015 PMC7479286 32939336

[29] Shi H; Li Y; Xu K; Yin J Advantages of photo-curable collagen-based cell-laden bioinks compared to methacrylated gelatin (GelMA) in digital light processing (DLP) and extrusion bioprinting. Mater. Today Bio 2023, 23, 100799. 10.1016/j.mtbio.2023.100799 PMC10519825 37766893

[30] Hsu HH; Kracht JK; Harder LE; Rudnik K; Lindner G; Schimek K; Marx U; Pörtner R A Method for Determination and Simulation of Permeability and Diffusion in a 3D Tissue Model in a Membrane Insert System for Multi-well Plates. J. Vis. Exp. 2018, 132, 56412. 10.3791/56412 PMC5931342 29553546

[31] Amar S; Smith L; Fields GB Matrix metalloproteinase collagenolysis in health and disease. Biochim. Biophys. Acta-Mol. Cell Res. 2017, 1864, 1940–1951. 28456643 10.1016/j.bbamcr.2017.04.015 PMC5605394

[32] Cho H; Kim J; Ahn JH; Hong YK; Mäkinen T; Lim DS; Koh GY YAP and TAZ Negatively Regulate Prox1 during Developmental and Pathologic Lymphangiogenesis. Circ. Res. 2019, 124, 225–242. 30582452 10.1161/CIRCRESAHA.118.313707

[33] Yao Y; Zaw AM; Anderson DEJ; Jeong Y; Kunihiro J; Hinds MT; Yim EKF Fucoidan and topography modification improved in situ endothelialization on acellular synthetic vascular grafts. Bioact. Mater. 2023, 22, 535–550. 36330164 10.1016/j.bioactmat.2022.10.011 PMC9619221

[34] Abu Taha A; Schnittler H-J Dynamics between actin and the VE-cadherin/catenin complex. Cell Adhes. Migr. 2014, 8, 125–135. 10.4161/cam.28243 PMC4049858 24621569

[35] Hooks JST; Bernard FC; Cruz-Acuña R; Nepiyushchikh Z; Gonzalez-Vargas Y; García AJ; Dixon JB Synthetic hydrogels engineered to promote collecting lymphatic vessel sprouting. Biomaterials 2022, 284, 121483. 35428014 10.1016/j.biomaterials.2022.121483 PMC9134840

[36] Kim S; Chung M; Jeon NL Three-dimensional biomimetic model to reconstitute sprouting lymphangiogenesis in vitro. Biomaterials 2016, 78, 115–128. 26691234 10.1016/j.biomaterials.2015.11.019

[37] Ahmadzadeh N; Robering JW; Kengelbach-Weigand A; Al-Abboodi M; Beier JP; Horch RE; Boos AM Human adipose-derived stem cells support lymphangiogenesis in vitro by secretion of lymphangiogenic factors. Exp. Cell Res. 2020, 388, 111816. 31923426 10.1016/j.yexcr.2020.111816

[38] Song JW; Munn LL Fluid forces control endothelial sprouting. Proc. Natl. Acad. Sci. USA 2011, 108, 15342–15347. 21876168 10.1073/pnas.1105316108 PMC3174629

[39] Henderson AR; Ilan IS; Lee E A bioengineered lymphatic vessel model for studying lymphatic endothelial cell-cell junction and barrier function. Microcirculation 2021, 28, e12730. 34569678 10.1111/micc.12730 PMC9274261

[40] Henderson AR; Choi H; Lee E Blood and Lymphatic Vasculatures On-Chip Platforms and Their Applications for Organ-Specific In Vitro Modeling. Micromachines 2020, 11, 147. 32013154 10.3390/mi11020147 PMC7074693

[41] Lammert A; Goldstein L; Narayanan S; Iskarous K Statistical methods for estimation of direct and differential kinematics of the vocal tract. Speech Commun. 2013, 55, 147–161. 24052685 10.1016/j.specom.2012.08.001 PMC3774006

