# Supplementary material for: Characterization of Photo-Crosslinked Methacrylated Type I Collagen as a Platform to Investigate the Lymphatic Endothelial Cell Response
Source: Lymphatics. Author manuscript; Available in PMC 2024 Dec 11. (PMC11632916; doi:10.3390/lymphatics2030015)
Supplement: supplementary material [file NIHMS2024373-supplement-supplementary_material.zip › lymphatics-3121598-supplementary.pdf]

Supplementary Data

Characterization of Photo-Crosslinked Methacrylated Type I Collagen as a Platform to Investigate the Lymphatic Endothelial Cell Response

Brian N. K. Ruliffson [1], Stephen M. Larson [1], Eleni K. Xhupi [1], Diana L. Herrera-Diaz [2] and Catherine F. Whittington [1,*]

Supplementary Data

The cytotoxicity of photoinitiator ruthenium and sodium persulfate (Ru/SPS) was assessed through a live/dead viability study. Three experimental groups were used to examine the effects of different conditions on Human Dermal Lymphatic Endothelial cells (HDLEC; PromoCell). All groups used methacrylated collagen (PhotoCol®; 8 mg/mL; Advanced BioMatrix) coated with fibronectin and were seeded with HDLECs at 8,000 cells per well. One group was prepared with photoinitator (Ru/SPS) within PhotoCol® and photo-crosslinked for 90 seconds (405 nm light) to generate a stiff matrix. One group was prepared with PhotoCol® and Ru/SPS but remained uncrosslinked to generate a soft matrix. The final control group was uncrosslinked PhotoCol® without Ru/SPS. All samples were cultured for 7 days prior to staining with Live/Dead assay (CalceinAM – live; Ethidium homodimer - dead) (ThermoFisher Scientific) and imaged immediately on Keyence BZX810 All-in-One Fluorescence Microscope (KEYENCE Corp. of America) (4X magnification). A total cell count was performed using ImageJ to calculate the percentage of living and dead cells.

Figure S1. Viability of HDLECs seeded upon PhotoCol® with Ru/SPS

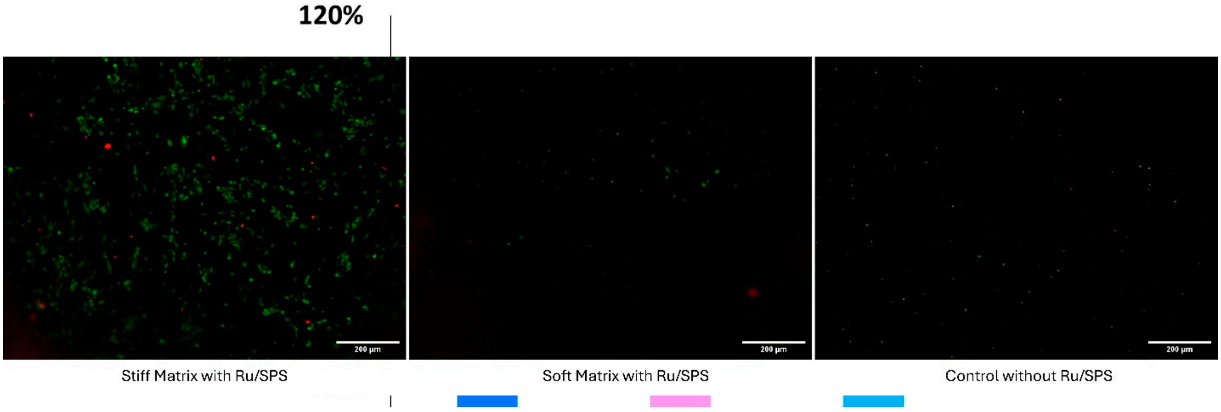

Figure S2: **Live/Dead representative images of HDLECs seeded upon PhotoCol® with Ru/SPS**. HDLECs were seeded upon: Stiff matrix (90 seconds photo-crosslinked; 6 kPa), Soft matrix (uncrosslinked; 0.5 kPa) and an uncrosslinked control without Ru/SPS. All surfaces were coated with fibronectin. Green represents live cells (CalceinAM) and red represents dead cells (Ethidium homodimer). Scale bars represent 200 µm.

Figure S1: Viability of HDLECs Seeded upon PhotoCol® with Ru/SPS. The ratio of live HDLECs to total number of HDLECs was used to calculate live cell percentage on: Stiff matrix (90 seconds photo-crosslinked; 6 kPa), Soft matrix (uncrosslinked; 0.5 kPa) and an uncrosslinked control without Ru/SPS. All surfaces were coated with fibronectin. Bars represent mean+SD (N= 4).

Figure S2. Live/Dead representative images of HDLECs seeded upon PhotoCol® with Ru/SPS
