# Supplementary figures and images for: Characterization of Photo-Crosslinked Methacrylated Type I Collagen as a Platform to Investigate the Lymphatic Endothelial Cell Response
Source: Lymphatics. Author manuscript; Available in PMC 2024 Dec 11. (PMC11632916; doi:10.3390/lymphatics2030015)

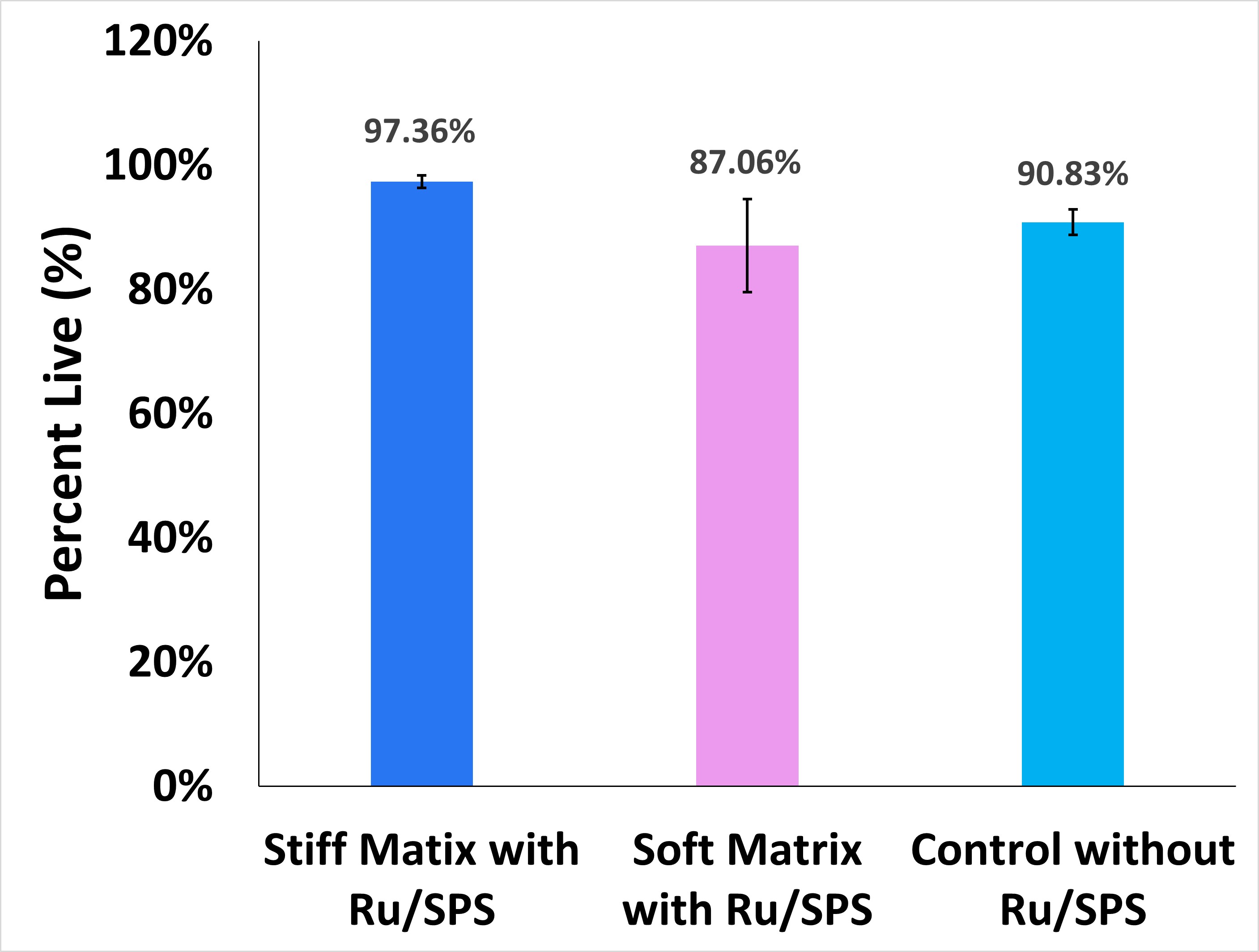

Supplement: supplementary material [file NIHMS2024373-supplement-supplementary_material.zip › Figure S1.jpg]

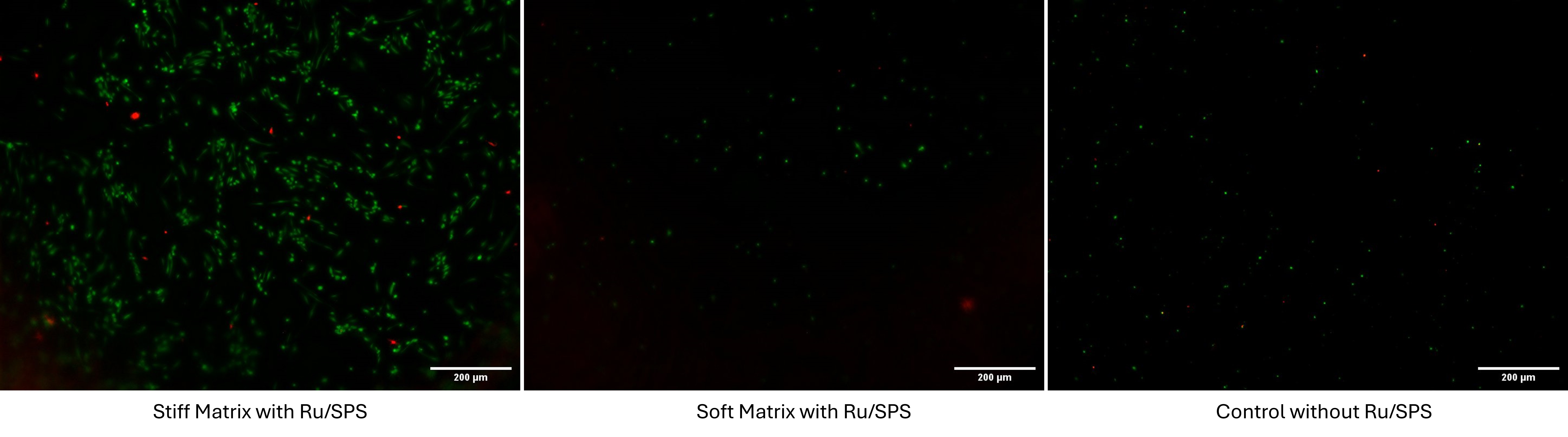

Supplement: supplementary material [file NIHMS2024373-supplement-supplementary_material.zip › Figure S2.jpg]
